# Acetobixan, an Inhibitor of Cellulose Synthesis Identified by Microbial Bioprospecting

**DOI:** 10.1371/journal.pone.0095245

**Published:** 2014-04-18

**Authors:** Ye Xia, Lei Lei, Chad Brabham, Jozsef Stork, James Strickland, Adam Ladak, Ying Gu, Ian Wallace, Seth DeBolt

**Affiliations:** 1 Department of Horticulture, University of Kentucky, Lexington, Kentucky, United States of America; 2 Department of Biochemistry and Molecular Biology, Pennsylvania State University, State College, Pennsylvania, United States of America; 3 United State Department of Agriculture Forage-Animal Production Research Unit, University of Kentucky Campus, USDA-ARS, Lexington, Kentucky, United States of America; 4 Waters Waters Corporation, Milford, Massachusetts, United States of America; 5 Department of Biochemistry and Molecular Biology, University of Nevada, Reno, Nevada, United States of America; Iowa State University, United States of America

## Abstract

In plants, cellulose biosynthesis is an essential process for anisotropic growth and therefore is an ideal target for inhibition. Based on the documented utility of small-molecule inhibitors to dissect complex cellular processes we identified a cellulose biosynthesis inhibitor (CBI), named acetobixan, by bio-prospecting among compounds secreted by endophytic microorganisms. Acetobixan was identified using a drug-gene interaction screen to sift through hundreds of endophytic microbial secretions for one that caused synergistic reduction in root expansion of the leaky At*cesA6^prc1-1^* mutant. We then mined this microbial secretion for compounds that were differentially abundant compared with *Bacilli* that failed to mimic CBI action to isolate a lead pharmacophore. Analogs of this lead compound were screened for CBI activity, and the most potent analog was named acetobixan. In living Arabidopsis cells visualized by confocal microscopy, acetobixan treatment caused CESA particles localized at the plasma membrane (PM) to rapidly re-localize to cytoplasmic vesicles. Acetobixan inhibited ^14^C-Glc uptake into crystalline cellulose. Moreover, cortical microtubule dynamics were not disrupted by acetobixan, suggesting specific activity towards cellulose synthesis. Previous CBI resistant mutants such as *ixr1-2, ixr2-1* or *aegeus* were not cross resistant to acetobixan indicating that acetobixan targets a different aspect of cellulose biosynthesis.

## Introduction

Biologically active small molecules are extremely useful tools that facilitate the dissection of cellular pathways in a manner that is often unattainable by genetic methods. These compounds can overcome genetic redundancy by acting on multiple protein targets and can be applied at defined times or concentrations to circumvent the use of potentially lethal loss-of-function mutations. The capacity to identify synthetic bioactive compounds has been aided by advancements in high-throughput screening platforms as well as combinatorial chemical libraries [1]. These approaches have been employed by a community of researchers to identify compounds that interfere with plant metabolic processes [2]–[7], signal transduction pathways [8]–[12], and vesicle trafficking events [13]–[15]. Despite their importance, the synthetic combinatorial libraries used to identify many of these compounds were constructed within the known limitations of chemical synthesis. However, naturally synthesized products are not subject to these limitations and represent an underexploited frontier of chemical diversity. Furthermore, it has been estimated that approximately two-thirds of the useful chemicals identified in the past quarter century were derived from secondary metabolites found in nature [16]. However, identification of useful lead compounds from complex biological samples remains challenging due to the fact that bioactive small molecules must be purified away from numerous compounds that do not confer the activity of interest.

Cellulose biosynthesis inhibitors (CBIs) represent one of the many successful examples of metabolic manipulation via small molecule inhibition in plants. Cellulose is the most abundant biopolymer on Earth, and this crystalline polysaccharide fundamentally influences plant cell shape and morphogenesis [17]. Cellulose is synthesized at the plasma membrane by cellulose synthase A (CesA) proteins [18]–[21], which serve as catalytic subunits in a large protein complex termed the “rosette”. Inhibition of cellulose biosynthesis induces loss of anisotropic expansion, radial cell swelling, and acute inhibition of plant growth [22]. Using these phenotypes as a proxy, a number of synthetic CBIs have been isolated, including isoxaben, quinoxyphen, dichobenil (DCB), CGA 325'615, and AE F150944 [23]–[28]. Thaxtomin A, which is also a potent inhibitor of cellulose biosynthesis [27], was characterized as a secondary metabolite isolated from the plant pathogen *Streptomyces scabies* [29]–[30], suggesting that some plant-interacting microorganisms have the capacity to produce CBIs.

Chemical genomics and cell biological studies have indicated that many known CBIs directly influence CesA function. Live-cell imaging of fluorescently-labeled CesA complexes in Arabidopsis seedlings treated with isoxaben, quinoxyphen or thaxtomin A revealed that these small molecules alter the localization of the CesA complex from active plasma membrane-localized particles to microtubule-associated compartments (MASCs; SMaCCs) underlying the plasma membrane [28], [31]–[34]. In contrast, DCB treatment completely inhibited CesA particle movement at the plasma membrane, suggesting a different mode of action for DCB [35]. Forward genetic screens for resistance to these CBIs suggest that many of these compounds may directly target CesA proteins. For example, an Arabidopsis screen for seedlings resistant to isoxaben identified two loci (*ixr1-1* and *ixr2-1*) that were mapped to mutations in CesA3 and CesA6, respectively [34], [36]. Similarly, a quinoxyphen resistant mutation (*ags*) was mapped to an A-V missense mutation in the C-terminus of CesA1 and the experimental structure of bacterial cellulose synthase suggests that this residue is directly involved in glucan contact during cellulose chain translocation [37]. The current investigation aimed to identify compound(s) capable of cellulose biosynthesis inhibition.

## Methods and Materials

### Endophyte isolation

Switchgrass plants were collected separately in July 2010 from two reclaimed strip-mining sites in western Kentucky (USA), where they were established as a monoculture during reclamation approximately 20 years ago. Shoot (leaves and stems) and root segments of approximately 1–1.5 cm in length were hand cut from collected switchgrass plants. These segments were sequentially washed with deionized water to remove soil and debris, rinsed with 95% (v/v) ethanol for 2 minutes, and then immersed in a solution of 30% (v/v) household bleach for 20 minutes. The segments were washed five times in sterile water and placed on separate YPDA-agar medium plates (2% [w/v] peptone, 1% [w/v] yeast extract, 2% [w/v] glucose, 0.003% [w/v] adenine hemisulfate) supplemented with 100 µg/mL Nystatin to prevent fungal growth. The plates were then incubated for 3–5 days in a growth chamber at 26°C. Single colonies arising from these plates were cultured separately and are reported in full [38].

### DNA extraction and 16S rDNA sequence analysis

Individual colonies were grown separately in YPDA liquid media at 26°C for 24 hrs on a rotary shaker at 200 rpm. Bacterial cultures were centrifuged at 3000× g for 10 min to pellet cells. Genomic DNA was extracted using a Zymo Research fungal/bacterial DNA miniprep kit (Zymo Research) following the manufacturers' instructions. The 16S rDNA region of each strain was amplified by PCR in a 50 µL reaction containing 3 µL of template genomic DNA, Taq DNA polymerase, and gene-specific primers 27f (5′-GAGTTTGATCCTGGCTCA-3′) and 1498r (5′-ACGGCTACCTTGTTACGACTT-3′), which are complimentary to the conserved regions at the 5′- and 3′- ends of the *E. coli* 16S rDNA gene at nucleotide positions 9–27 and 1477–1498, respectively [39]. PCR amplification was performed using a Bio-Rad iCycler with the following PCR conditions: initial denaturation of 94°C for 5 min, followed by 50 cycles of 94°C for 1 min, 54°C for 1 min, and 72°C for 2 min with a final extension of 72°C for 5 min. The PCR products were purified using a Fermentas GeneJET PCR purification kit (Fermentas Inc., MD, USA) and sequenced by ELIM BioPharm Inc. The sequences were analyzed using the BioEdit Sequence Alignment Editor (http://www.mbio.ncsu.edu/BioEdit/bioedit.html) and were subjected to BLASTn searches in the NCBI and BIBI Databases [40], [41] for microbial identification.

### Bacterial secreted extract preparation and cellulose biosynthesis inhibitor screen

Isolated bacterial colonies were grown separately in 100 mL of YPDA broth at 26°C on a rotary shaker at 200 rpm for 2 weeks, and the bacterial cells were removed by centrifugation at 3000 rpm for 10 min. Culture supernatants were collected, freeze dried by lyophilization, dissolved in 1 mL of sterile deionized water, and immediately stored at −80°C.

The resolubilized bacterial extracts were assayed for their ability to synergistically inhibit root elongation of the Arabidopsis cellulose synthesis mutant *procuste1-1* (*cesA6^prc1-1^*)[19] in a plate-based assay. Wild-type Col-0 and *cesA6^prc1-1^* seeds were surface sterilized in 30% (v/v) household bleach and 0.1% (w/v) sodium dodecyl sulfate for 15 min at 25°C, followed by thorough rinsing with sterile deionized water. Seeds were stratified for 4 days at 4°C to synchronize germination and plated on MS-agar media (1/2 strength Murashige and Skoog salts, 1% [w/v] sucrose, 1% [w/v] phytoagar, pH 5.7) supplemented with 50 µL of solubilized bacterial secreted extracts. MS-agar medium plates with no addition of bacterial extract served as negative controls. Seeds were plated horizontally with each plate containing at least ten seeds of each genotype, and the plants were grown in an incubator under long-day conditions (16 hr light/8 hr dark) for 7–9 days at 22°C. Root lengths for each plant genotype were measured using ImageJ software, and the additive inhibition of *cesA6^prc1-1^* root growth was calculated. Extracts from bacteria that synergistically inhibited *cesA6^prc1-1^* were identified and subjected to further analysis.

### Identification of differentially abundant metabolites by UPLC chromatography and mass spectrometry

Resolubilized bacterial secretions were diluted 1∶50 in water, and 10 µL of these solutions were injected into an Acquity UPLC BEH C18 2.1×100 mm 1.7 µm particle column. Small molecules were separated using mobile phase buffer A (10 mM ammonium acetate, 0.1% [v/v] formic acid) and buffer B (10 mM ammonium acetate, 0.1% [v/v] formic acid, 100% acetonitrile) with the following gradient conditions: initial (95-5 A-B), 0–0.5 min (95-5 A-B), 0.5–7 min (5-95 A-B), 7–9 min (5-95 A-B), 9–9.01 (95-5 A-B) and 9.01–10 (95-5 A-B). The gradient flow rate was 500 mL/min. Mass spectrometry conditions included the following parameters: acquisition mode was Resolution mode MS^E^, positive and negative ion modes, scan speed of 0.1 seconds/scan, source temperature of 120°C, desolvation temperature of 650°C, desolvation gas (1000 L/hr), capillary voltage of 1 kV, cone voltage of 20 V, extraction cone voltage of 4 V, cone gas (10 L/hr), resolution 20,000 (FWHM), a mass range of 50–1200 Da, and high energy collision ramp (20–35 eV). Processing of the data sets included metabolic subtraction of *B. sp.-B* peaks from the *B. sp.-A* spectrum using the MarkerLynx XS software (Waters Milford MA). The MarkerLynx software generated a database of differentially abundant biomarker compounds indexed by retention time and exact mass, which was used to create a results matrix. MassFragment software (Waters, Milford MA) used the input mass of a differentially abundant compound and the product ions to generate predicted lead compound structures. The PCA was carried out with the data of all samples. The UPLC-MS data were processed by MarkerLynx XS and EZinfo, sub modules of the MassLynx 4.1 software (Waters, Milford, MA) to accomplish signal deconvolution and MVA. The intensity threshold was set to 1000 counts. Using the EZinfo module, OPLS-DA was carried out with UPLC-MSe data of 4 samples, at least 5 replicates of each sample in both electrospray positive and negative mode to discover relatively important chemical markers. The data scaling method was Pareto.

### Chemical analogs

Chemical analogs for the lead optimization screen were purchased from Sigma Aldrich (St Louis, MO) and Chembridge Corporation (San Deigo, CA).

### Confocal microscopy imaging of cellulose synthase complexes

The construction of transgenic Arabidopsis plants expressing GFP-CesA3 or YFP-CesA6 under their native promoters, dual-labeled lines expressing GFP-CesA3 and 35S:: mCherry-TUA5, and GFP-PIP2 were previously described [20], [31], [33], [42]. Seeds were surface sterilized and stratified as described above and were plated on MS-agar medium lacking sucrose. Seedlings were grown vertically in the dark at 22°C for 3 days.

For microscopic observations, seedlings were mounted in water between 24×60 mm glass slides and 22×22 mm cover slips separated by vacuum grease spots as previously described [31]. For drug treatments, the mounting solution was supplemented with 50 µM test compound and incubated for 1 hr in darkness at 25°C prior to imaging. Seedlings treated with 0.25% (v/v) DMSO served as negative controls. The seedlings were observed using a Leica SD6000 inverted confocal microscope system featuring a 100X/1.4 NA oil immersion objective, a Yokogawa CSU-X1 spinning disk head, 488 and 561 nm lasers, and Metamorph control software (Molecular Devices). Z-series images were collected at a step size of 200 nm and analyzed using ImageJ software (http://rsbweb.nih.gov/ij/).

### Small molecule modeling

Comparative structural analysis of acetobixan and isoxaben was performed using the Molecular Operating Environment (MOE) software (Chemical Computing Group; Montreal, Quebec, Canada). Molecules were constructed using the Builder module and subjected to energy minimization using the MMFF94x forcefield and an RMS gradient of 0.05 Å. After each molecule was prepared, they were structurally superimposed using the Flexible Alignment algorithm under the default settings for the iteration limit and energy cutoff. This algorithm performs a stochastic conformational search to find the best low energy conformations that maximize structural as well as chemical overlap between two molecules and reports the results in a database format. The conformational superposition with the lowest potential energy was used for further analysis.

### Cellulose content measurements

Total cellulose content was measured in 5-day-old dark grown Arabidopsis seedlings by the method of Updegraff [43]. Briefly, seedlings were treated with 70% (v/v) ethanol for 1 hr at 70°C, then transferred to 1∶1 chloroform/methanol solution for 4 hrs, followed by an acetone wash. The residue was air dried for 48 hrs and weighed. Cellulose content was determined colorimetrically using anthrone reagent. To assess the biochemical effects of cellulose biosynthesis inhibitors, cellulose biosynthesis rates were directly estimated by [^14^C]-glucose incorporation assays. Arabidopsis seedlings were grown in the dark for 3 days in liquid MS media supplemented with 2% (w/v) dextrose. The seedlings were washed in dextrose-free liquid MS media and blotted dry. Twenty milligrams of total wet weight seedling biomass were transferred to 0.5 mL of dextrose-free MS liquid media in 1.5 mL eppendorf tubes. The seedlings were centrifuged and the supernatant media was removed and replaced with 0.5 mL of dextrose-free MS liquid media supplemented with 1 µCi/mL of ^14^C glucose. For inhibitor treatments, seedlings were treated with either 100 nM isoxaben or 20 µM acetobixan. Seedlings treated with 0.01% (v/v) DMSO served as negative controls. Seedlings were incubated in the dark for 1 hr, centrifuged, and washed 3 times in dextrose-free MS liquid media to remove excess radiolabeled glucose. The remaining plant material was boiled in acetic nitric acid reagent [43] for 30 minutes, cooled to 25°C, and then centrifuged for 5 minutes to pellet the insoluble material. Four hundred µL of supernatant was removed and placed in a separate vial for further analysis. The remaining liquid as well as the insoluble material was washed three times (in dH_2_0), resuspended in 0.5 mL of dextrose-free MS liquid media, and transferred to a scintillation vial containing 5 mL of scintillation cocktail. The radioactivity of these samples and the supernatants remaining after digestion of the biomass by acetic nitric acid reagent were quantified by liquid scintillation counting.

## Results

### Identification of indexed bacterial endophytes capable of inhibiting cellulose biosynthesis

Cross-kingdom signaling between endophytes and the plant host has been demonstrated to depend on small molecules in some systems [44]. Since the cell wall represents a key barrier to the entry of endophytic microorganisms into the plant, it is plausible that some endophytic species may secrete small molecules that reversibly inhibit the synthesis of cell wall polysaccharides to temporarily weaken the cell wall and facilitate microbial entry. To test this hypothesis, we examined the ability of small molecule secretions derived from a library of switchgrass (*Panicum virgatum L.*) endophytes to synergistically inhibit root growth of the *cesA6^prc1-1^* mutant. The *cesA6^prc1-1^* mutant was used because other known CBIs, such as isoxaben, exhibit a synergistic effect on root expansion [45]. Therefore, the *cesA6^prc1-1^* mutant was used purely as a screening tool to prioritize candidate endophyte secretions for putative CBI activity.

**Figure 1:**
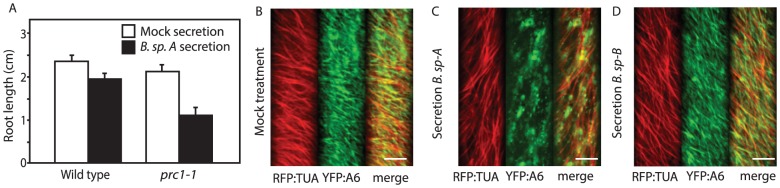
Screening of microbial extracts for putative CBI compounds. A) Wild-type Col-0 and *cesA6^prc1-1^* seedlings were grown in the presence of mock secretion (white bars) or *B. sp.-A* secretion (black bars) for seven days in constant light at 22°C. Seedling primary root lengths were quantified using ImageJ (Error bars represent SEM; n>10). Live-cell imaging of YFP::CESA6, RFP::TUA in cells treated with mock (B), *B. sp*. -A secretion (C) and *B. sp*. -B secretion (D). Images represent time-averaged projections of 61 frames spaced 5 s apart. Movement of mock-treated YFP::CESA6 gives rise to strands of YFP emission (B and D), whereas the crude secretion clears the YFP::CESA6 label from the plasma membrane focal plane after 4 hr (C). Scale bars  = 10 µm.

Only endophytic microorganisms that could be cultured in isolation were considered for this study. Individual isolates were grown in rich media, and the resulting culture supernatants were concentrated by lyophilization. Small molecules in the culture supernatants were resuspended and added to plant growth medium to determine if these secretions had any effect on wild-type or *cesA6^prc1-1^* root growth, and the synergistic inhibition of *cesA6^prc1-1^* root growth was calculated by comparing the relative inhibition of expansion between *cesA6^prc1-1^* mutant and wild-type. In a screen of 200 microbial secretions, one was identified that inhibited *cesA6^prc1-1^* root growth by 57% greater than wild-type plants (**Fig. 1A**). As expected, the majority of secretions elicited no effect on growth of either *cesA6^prc1-1^*or wild-type plants. To determine the identity of the organism secreting active CBIs, we sequenced the 16S rDNA intergenic region and identified the organism as a member of the *Bacillus* genus (referred to for the remainder of the manuscript as *B. sp.-A*).

### *B. sp-A* crude secretions mimic the effects of known cellulose biosynthesis inhibitors

Based on the design of the primary chemical genetics screen, it was evident that the *B. sp.-A* crude secretion inhibited expansion of *cesA6^prc1-1^* seedlings more than wild-type. Live-cell imaging of fluorescently-tagged CesA subunits is a useful tool to investigate defects in cellulose synthesis during chemical or genetic disruption [31], [35]. Furthermore, a functional association between cortical microtubules and cellulose synthase has been established [31], therefore we performed our drug experiments using a double tagged line expressing both YFP-CesA6 and RFP-TUA5. Upon treatment with freeze-dried secretion derived from *B. sp.-A* the YFP-CesA6 punctae that are normally at the plasma membrane cleared from the membrane and became cytoplasmic (**Fig 1B-C**). No observable defects in microtubule motility or morphology were induced (**Fig 1C**, a representative image from 16 cells of 10 individual seedlings, **Movie S1 and File S1**). A clearance of YFP-CesA6 was previously identified for the CBI isoxaben [31]. Isoxaben also caused YFP-CesA6 fluorescent signal to accumulate in intracellular small microtubule associated compartments SmaCCs/MASCs [32]–[33], which was also documented upon *B. sp.-A* treatment (**Movie S1 and File S1** for evidence of CESA particle tracking on the minus end of a cortical microtubule). Mock treated seedlings displayed typical steady velocity of CesA particles at the plasma membrane (**Fig 1B**, a representative image from 8 cells of 5 individual seedlings).

### Subtractive metabolomics between taxonomically related *B. sp.* isolates with differential effects on cellulose biosynthesis

A strategy was sought to use subtractive metabolomics to identify differentially abundant compounds present in *B. sp.-A*, as possible lead compounds. However, we first needed a rational control secretion. This control secretion was identified by cross-referencing the endophyte-library for a bacteria with a similar 16S rDNA sequence to *B. sp.-A* that not cause synergistic inhibition of *cesA6^prc1-1^* root growth. We identified one *Bacillus* species [38] that displayed 98% homology to *B. sp.-A* in terms of 16S rDNA sequence but did not cause a CBI-like effect. We refer to this strain as *B. sp.-B.* As a further assessment of the negative properties of *B. sp.-B* secretion, transgenic Arabidopsis seedlings expressing YFP-CesA6 and RFP-TUA5 were treated with *B. sp.-B* secretion. This treatment failed to result in any noticeable clearance of YFP-CesA6 from the plasma membrane or any changes in the morphology and motility of cortical microtubule arrays (**Fig 1D**, a representative image from 15 cells of 6 individual seedlings, **Movie S2 and File S1**).

**Figure 2:**
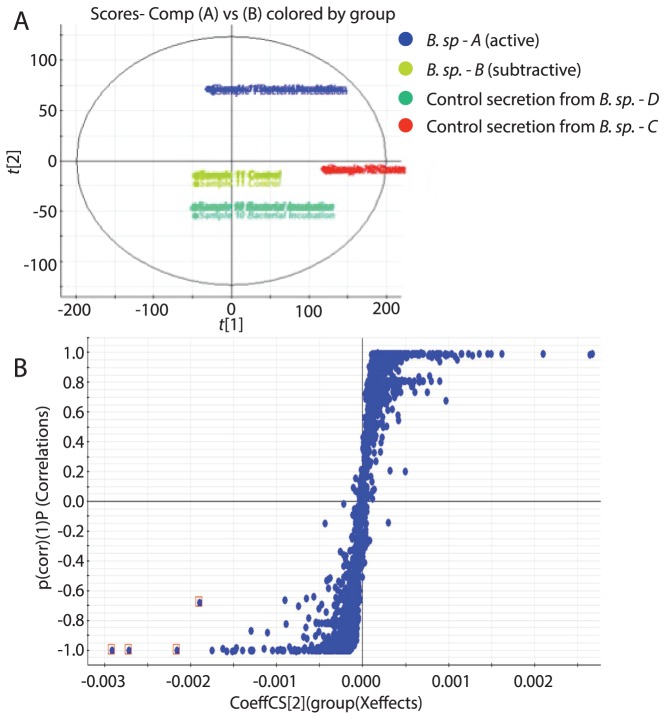
Subtractive metabolomics between microbial secretions with differential CBI activity. The metabolite profiles of *B. sp.-A* (blue), *B. sp.-B* (green), and two other CBI-inactive microbial secretions (red and aqua) were used to generate a PCA plot of secreted metabolite composition (A). Clustering of the replicates supported the separation of the two *B. sp*. strains and highlights the potential existence of one or more biomarkers that facilitate the characterization of the bioactive secretion components. An S-plot (B) was then used as a multivariate analytical technique to identify putative biomarkers by combining covariance and correlation measures for all analyses together in a scatter plot. Variables included in the matrix that separated compounds were exact mass, abundance and retention time. The y-axis indicates the confidence, or reliability of a measure and the x-axis reflects the magnitude of the variable. Note that features falling on the “tails” of the S-plots are most likely to be biomarkers that are differentially present in samples. Differentially abundant compounds which represent potential CBI compound candidates are highlighted in red boxes.

Subtractive metabolic profiling used a SYNAPT-G2 UPLC-MS system (Waters, Milford MA) to compare the chemical composition of *B. sp.-A* and *B. sp.-B* secretions, based on their retention time and mass spectrum. Replicate samples (n = 5) were first analyzed by principle component analysis (PCA), which indicated that secretions derived from *B. sp.-A* and *B. sp.-B* are clearly separated and that secretions derived from *B. sp.-B* were more similar to control secretions that did not produce synergistic inhibition of root length in cesA6*^prc1-1^*. The separation of the two B. sp. secretions in the PCA plot indicated that some chemical components between the two secretions varied and further suggested the existence of one or more chemical biomarkers that could facilitate the identification of the endogenous CBI compounds in the *B. sp.-A* secretion (**Fig 2A**). To further analyze the comparative metabolomic profiles of *B. sp.-A* and *B. sp.-B*, we generated an S-plot of their secreted metabolite profiles (**Fig 2B**). An S-plot is a multivariate analytical technique that relates the covariance and correlation measurements for all analyses together in a scatter plot, and this data representation is a useful tool for identifying putative biomarkers. It is useful to note that features which fall on the “tails” of S-plots are most likely to be biomarkers that are differentially present in all samples analyzed. The S-plot analysis of *B. sp.-A* and *B. sp.-B* secretions identified at least four potential biomarkers that were differentially produced by these two species and these compounds were selected for further structural analysis because they could be either bioactive CBIs or precursors to the causative molecule.

**Figure 3:**
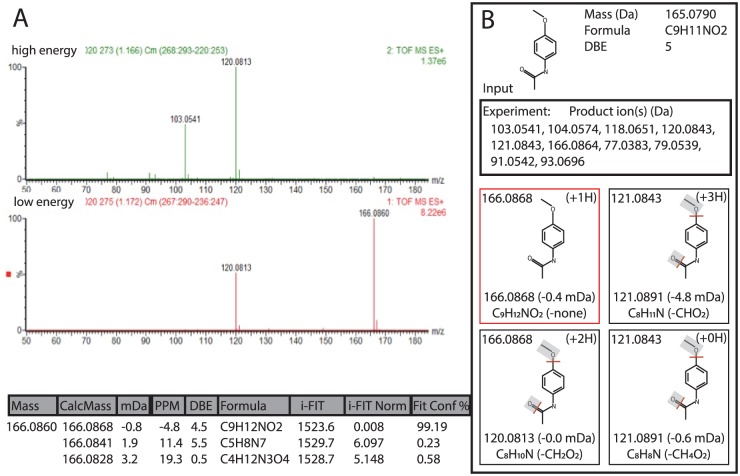
Identification of a CBI lead compound in *B. sp.-A* secreted extracts by mass fragment analysis. The mass fragmentation pattern of the differentially abundant compound was examined using high and low energy collisions, resulting in a fragmentation pattern that could be computationally interrogated using MassFragment software (Water Corporation, Baltimore MD)(A). The experiment presented involved the input of the mass fragmentation data from putative N-(4-methoxyphenyl)acetamide parent marker to determine the fit between the experimental data and computational prediction of fragmentation (B), which was over 99%.

To analyze potentially bioactive compounds, prominent markers were selected for further structural analysis. A 166.0868 Da product ion derived from the mass spectrum of differentially abundant metabolites from the *B. sp.-A* secretion was computationally examined for potential matching structures (**Fig 3A**). These data led to candidate markers and we focused on one compound that was putatively identified as N-(4-methoxyphenyl)acetamide (**Fig 3B**) based on mass spectrum alignment and fragmentation. These results suggest that the product ion at 166.0860 Da may be a structural component of the bioactive compound(s) in the *B. sp.-A* secretion that contribute to cellulose biosynthesis inhibition.

### Analogs of N-(4-methoxyphenyl)acetamide inhibit cellulose biosynthesis

Once a putative pharmacophore was identified from within the secretion, we focused our whole plant assays on wild-type plants rather than the *cesA6^prc1-1^* mutant, as *cesA6^prc1-1^* was used as a tool to screen through crude secretions. To explore the pharamocophore structure in greater detail, we identified eleven chemical analogs of N-(4-methoxyphenyl)acetamide that were available as a fee-for-service chemical and examined their influence on root expansion (**Table 1**). A 20 µM screening concentration was selected due to being comparable to known CBIs, such as DCB and morlin (46,47). Three analogs were identified selected based on their capacity to inhibit root expansion (more than 95%) and one of these, N-(2-methoxybenzyl)-2-(phenylthio)acetamide (**Table 1**), was selected for detailed investigation and subsequently referred to as “acetobixan”. The pharmacophore N-(4-methoxyphenyl)acetamide did not induce effects that are generally elicited by CBIs, including anisotropic cell expansion and severe reduction in root elongation, suggesting that N-(4-methoxyphenyl)acetamide may only represent a substructure of the actual bioactive compound.

**Table 1:** CBI activity for N-(4-methoxyphenyl) acetamide and structural analogues.

Identifier	% wild type	Structural formulae
1	100	0.1% dimethylsufloxide
2	100	2-chloro-N-(4-methoxy-phenol)-acetamide
3	96	N-(2-amino-4-methoxyphenol)acetamide
4	92	N-(4-methoxyphenyl)acetamide
5	89	2-[(4-methoxyphenyl)acetyl]-1,2,3,4,-tetahydroisoquinoline
6	88	2-[3-(4-methoxyphenyl)propanoyl]-1,2,3,4,-tetahydroisoquinoline
7	46	N-[2-methoxybenzyl)-2-[(4methylphenyl)thio]propanamide
8	33	1-{[(4-chlorophenyl)thio]acetyl}]-1,2,3,4,-tetahydroisoquinoline
9	20	N-[2-methoxybenzyl)-2-[(4methylphenyl)thio]acetamide
10	10	N-[2-methoxybenzyl)-4-phenylbutanamide
11	>5	2-[(4-chlorophenyl)thio]-N-(2methoxylbenzyl)acetamide
12	>5	2-[(4-chlorophenyl)thio]-N-(2methoxylbenzyl)propanamide
13	>5	N-(2-methoxybenzyl)-2-(phenylthio)acetamide

**Figure 4:**
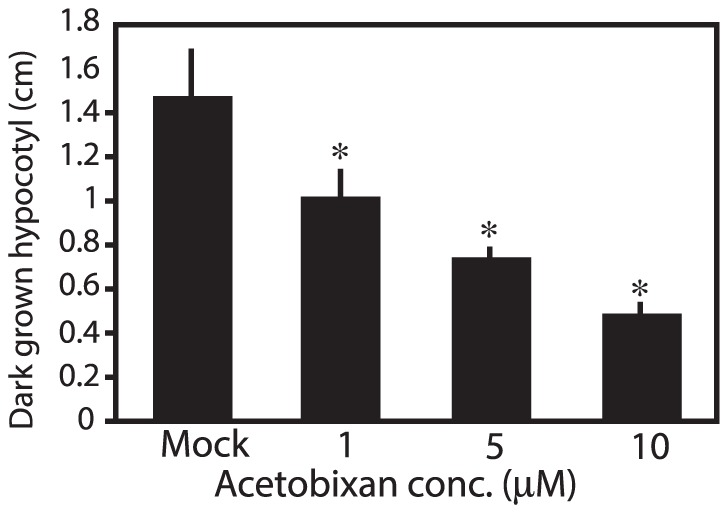
Acetobixan inhibited expansion in hypotocyl length, independent of light. Wild type Col-0 Arabidopsis seedlings were grown vertically in darkness on MS agar media containing the indicated concentrations of acetobixan. Data illustrated concentration dependent inhibition of expansion. Hypocotyl length was quantified using ImageJ (NIH, Bethesda, MD).

Acetobixan reduced expansion in a concentration dependent manner in light (not presented) or dark growth conditions (**Fig 4**). Dark conditions are presented as these data demonstrate that phytotoxic effects of acetobixan were independent of light. Light independent results exclude several alternative herbicidal modes of action that are enhanced by light such as photosynthesis, chlorophyll, and pigment inhibitors as the activity of acetobixan. Wild type seedlings grown on acetobixan-supplemented media displayed severe radial cell swelling and long-term treatment resulted in chlorosis as well as seedling death (**Fig S1**), as seen for other CBI structures [35]. While assays were focused on wild type seedlings, we anticipated that the *cesA6^prc1-1^* mutant would be hypersensitive to acetobixan. Results confirmed *cesA6^prc1-1^* hypersensitivity to acetobixan at dose rates that failed to significantly inhibit wild type expansion (*500% more sensitive*). This syndrome of phenotypes was consistent with the effects of known CBIs, such as isoxaben, quinoxyphen, and DCB [46], [47].

### Acetobixan inhibits cellulose biosynthesis in Arabidopsis and acts via clearance of cellulose synthase complexes from the plasma membrane

**Figure 5:**
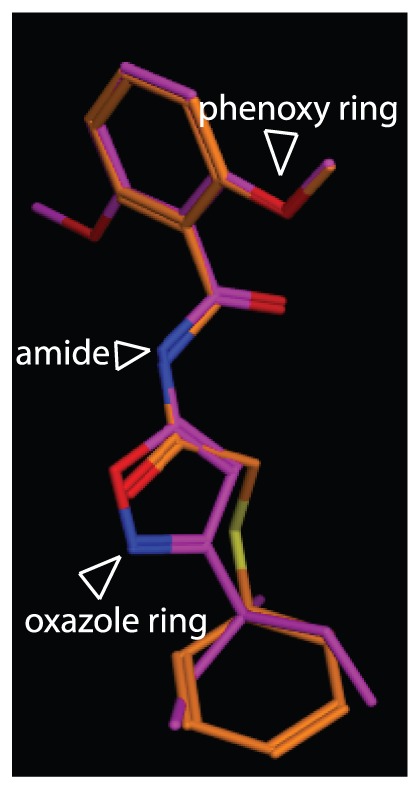
Acetobixan inhibits cellulose biosynthesis in Arabidopsis and causes clearance of CESA from the plasma membrane focal plane. (A) Cellulose content (as a percentage of dry weight) was measured by the anthrone method in 500 seedlings that had been germinated on MS agar media in tissue culture plates containing acetobixan at the concentrations indicated. Acetobixan-induced inhibition of 14C-glucose incorporation into the cellulosic fraction of plant cell wall polysaccharides (B). Analysis of YFP-CESA6 shows that acetobixan causes loss of CESA from the plasma membrane (C). Scale bar  = 10 µm.

To explore the CBI activity of acetobixan in greater detail we prepared acid insoluble residues from 7-day-old seedlings treated with 1–10 µM acetobixan and measured the cellulose content of these fractions (**Fig 5A**). This experiment demonstrated that acetobixan causes a concentration-dependent reduction in total cellulose content. The most robust assay of CBI activity to date is the of [^14^C]-glucose incorporation assay [46]. Here, over a short period of time (1 to 4 hr) the incorporation of [^14^C]-glucose into cellulose is measured with and without CBI treatment. Over a 1 hr period of exposure, acetobixan significantly reduced [^14^C]-labeled glucose incorporation into crystalline cellulose (**Table 2** **;** **Fig 5B**). To investigate whether the inhibition of cellulose biosynthesis was occurring via a similar mechanism as described for the original *B. sp.-A* secreted extract (**Fig 1C**), YFP-CesA6 expressing Arabidopsis seedlings were treated with acetobixan. This treatment resulted in rapid relocalization of cellulose synthase complexes from the plasma membrane into intracellular compartments (**Fig 5C**). Overall, these data strongly support the hypothesis that acetobixan is a cellulose biosynthesis inhibitor.

**Table 2:** The effects of acetobixan and isoxaben on the incorporation of ^14^C glucose into the cellulosic fraction of etiolated *Arabidopsis* seedling cell walls.

	Soluble fraction*	St Err**	Insoluble fraction*	St Err**
**DMSO**	100	5.1	100	4.3
**Acetobixan**	88.8	8.2	53.1	4.9
**Isoxaben**	83.1	10.8	48.6	3.5

*Results represent percentage of control.

**Standard error from the mean of three replicates.

### Resistance to acetobixan was not conferred by other CBI resistant mutants

**Figure 6:**
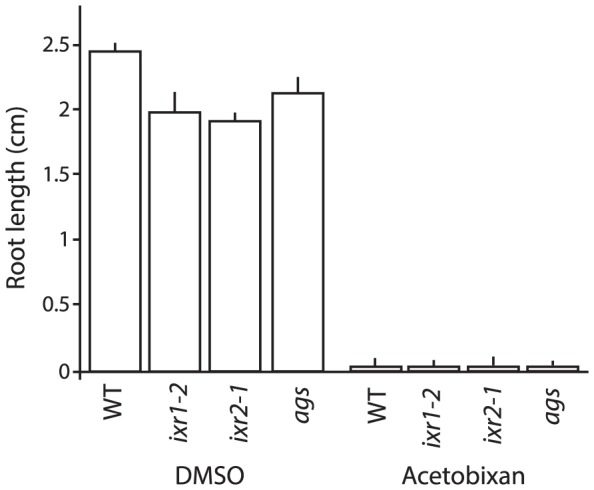
Isoxaben (*ixr1-2* and *ixr2-1*) nor quinoxyphen (*aegeus, ags*) resistant mutants displayed cross-resistance to acetobixan. Root length was measured in wild type and CBI resistant mutants grown vertically in constant light on MS media supplemented with mock or 20 µM acetobixan. Root lengths was measured after 7 d growth.

The isoxaben and quinoxyphen resistant mutants *ixr1-2, ixr2-1* and *aegeus* displayed no cross-resistance to acetobixan (**Fig 6**), suggesting that acetobixan targets a different component of the cellulose biosynthetic machinery than isoxaben or quinoxyphen.

### Structural modeling of acetobixan compared with known CBI

**Figure 7:**
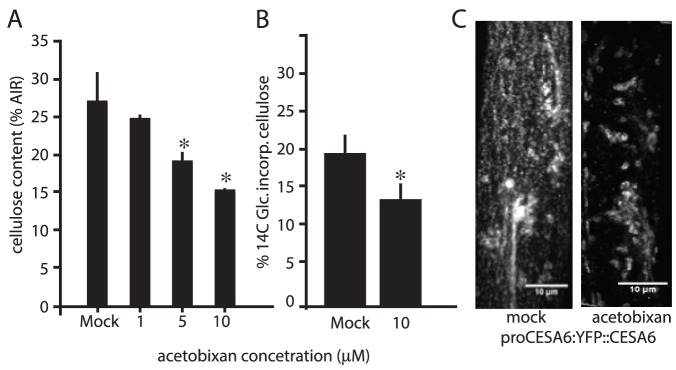
Acetobixan structural analysis. Acetobixan was experimentally overlaid with isoxaben using the Flexible alignment algorithm in the Molecular Operating Environment (MOE) software. Isoxaben is shown in purple and acetobixan is shown in gold. Oxygen atoms have been colored in red and nitrogen atoms in blue for convenience. The positions of the phenoxy ring, the amide moiety of acetobixan, and the oxazole ring of isoxaben are indicated with carats.

Since acetobixan represents a new member of a growing class of CBIs, we structurally compared acetobixan with other CBI structures and identified the only structure displaying any similarity was isoxaben. Superimposed acetobixan and isoxaben (via Flexible Alignment algorithm in the Molecular Operating Environment (MOE) software) (**Fig 7**) suggested that the phenoxy groups of both compounds were superimposable and that the ortho O-methyl group of acetobixan could be feasibly superimposed over either of the ortho O-methyl groups of isoxaben. This structural examination indicated that the ortho O-methyl group on these structures may be important for CBI bioactivity. These data agree with analog assessment data (**Table 2**) indicating that compounds exhibiting reduced bioactivity contain para O-methyl groups. Despite the capacity to superimpose parts of these two CBIs, as noted above, isoxaben resistant mutants were not cross-resistant to acetobixan. Notable differences in the superimposition analysis revealed the amide moiety in acetobixan overlapped with an oxazole ring of isoxaben. Further, the thioether moiety of acetobixan appears to function as a flexible spacer that links the amide portion of the molecule to the second phenyl ring. This structural analysis suggests that isoxaben and acetobixan share some similar chemical properties, but that structural differences between these molecules are significant enough to not confer cross-resistance.

## Discussion

Acetobixan caused radial swelling in *Arabidopsis* seedlings. The classical assay for CBI determination is by assessing the incorporation [^14^C]-labeled glucose into crystalline cellulose [46]. Upon exposure to acetobixan, incorporation [^14^C]-labeled glucose into crystalline cellulose was reduced. Cellulose accumulation was also inhibited in a dose dependent manner. Based on these findings, the mode of action of acetobian is consistent with its classification as a CBI. Cellulose synthesis is a complex process. Currently, it is thought that the CSC consists of 18–24 catalytic CESA proteins producing a strand of cellulose termed a microfibril that has a 7 nm^2^ cross sectional area [48]. Several necessary accessory proteins are required cellulose biosynthesis, such as KORRIGAN (endo-1,4-beta-d-glucanase)[49], CSI1 [50] and COBRA (glycosyl-phosphatidyl inositol-anchored protein)[51]. Despite a recent breakthrough in crystallizing a bacterial cellulose synthase [37], there are no robust *in vitro* assays for CSCs. Moreover, the bacterial cellulose synthase and plant CSCs have sufficient divergence that plants CBIs do not exhibit activity on bacteria [52], [37], [53]. Therefore, imaging fluorescently-tagged CesA subunits in living cells has been used to study how a CBI alters cellulose biosynthesis. These studies have in turn been useful to dissect the cortical cytoskeletons role in mediating the secretion and organized delivery of CESA to the plasma membrane [31], [54], [55]. Furthermore, accessory proteins to the core CESA subunit rosette complex, such as POM2/CELLULOSE SYNTHASE INTERACTING1 protein respond to CBIs in a parallel manner to CESA, suggesting the tight association between these proteins [52], [56]. In two cases, resistant mutants to CBI drugs (isoxaben and quinoxyphen) have encoded missense mutations in the CESA proteins, which have led to identifying fundamental aspects of the cellulose synthesis process, such as the link between crystallization and polymerization [28]. CBI resistant mutants have also been a source of invaluable functional mutations within the biochemically recalcitrant CESA to populated tertiary model structures of CESA [53]. With only a handful of drugs available to dissect cellulose synthesis, more are needed. The identification of acetobixan provides an additional tool.

Similar to several other CBI compounds, including isoxaben, thaxtomin A, AE F150944, CGA 325'615, and quinoxyphen [28], [33], [54], [57], acetobixan caused clearance of the CesA complex from the plasma membrane focal plane in living Arabidopsis seedlings (**Fig 5**). Despite commonality of clearance mechanism, resistant mutants for quinoxyphen [28] or isoxaben [34], [36] revealed no cross-resistance to acetobixan. These data suggest that these molecules may differentially affect cellulose biosynthesis and that target(s) for acetobixan may identify unique aspects of synthesis.

All known CBIs, including acetobixan in this study, have been identified by forward screening approaches that utilize synthetic small molecule libraries to find compounds that mimic a certain phenotype. We hypothesized that plant associated microorganisms may secrete natural products that are capable of modifying plant cellulose biosynthesis, and that these organisms could be systematically exploited to identify new small molecules. The implementation of two primary screens aided in the identification of microorganisms producing CBIs and subtractive metabolomics facilitated the identification of a pharmacophore. While quite an intriguing means to isolate a new drug, the active component of the CBI-active secretion remained elusive. Nonetheless, the identity of a *Bacilli* capable of inhibiting plant cellulose synthesis was interesting. The CBI Thaxtomin A is also a natural CBI, produced by *Streptomyces* species pathogenic to potato and other taproot crops [27]. As cellulose is both essential for plant cellular expansion and the most abundant carbon polymer synthesized by the plant, it is highly plausible that CBIs are produced by numerous microorganisms.

In our subtractive metabolic fingerprinting experiment, the Markerlynx software (Waters, Millford MA) was used to compare the metabolite data by considering both the chemical properties and abundance of each molecule to generate an S-plot of biomarker data (**Fig 2B**). Because the differential abundance of the compounds can be considered, we expect that this streamlined the subtractive nature of the experiment. It is also likely that this approach may be more broadly applicable for the identification of other biologically relevant small molecules, since secondary metabolite biosynthetic pathways and regulons in bacteria are often organized into operons [58], [59] which are differentially present in closely related bacterial species [60], [61]. Alternative approaches to identify a drug, such as fractionation and isolation, are also fraught with technical challenges [62], but are needed to narrow the potential scope of lead compounds from thousands of molecules to a manageable subset pharmacophore.

It is notable that the identified microbial secretion containing an active CBI was a member of the genus *Bacillus* (*B. sp.-A*). *Bacilli* are spore-forming, gram-positive bacteria that are widely distributed in aerobic terrestrial [63] and marine environments [64]. Numerous members of this genus have been identified as plant endophytic organisms [64]-[70]. Furthermore, secondary metabolite production among *Bacillus* species is common and secreted compounds with antibacterial, antifungal, hemolytic, photoprotective, iron acquisition-assisting and bacteriolytic activities have been identified [71]. Two possibilities exist to explain the capacity of *B. sp.-A* to synergistically alter cellulose synthesis through a drug-gene interaction with *procuste*. It is plausible that *B. sp.-A* either secretes CBI compounds due to its endophytic association with the host plant, or that it secretes such a compound only under physiologically abnormal conditions induced by isolated *in vitro* growth in media. Further investigation into the biology of this Bacilli are needed, as a biologically mediated *in situ* delivery mechanism for a CBI would be of interest.

## Supporting Information

Figure S1 Wild type Arabidopsis seedling treated with 20 µM acetobixan for 5 days displays radial cellular swelling (scale bar  = 1 mm).
(JPG)

File S1 Outlines experimental procedures used to generate Movie S1 and S2.
(DOCX)

Movie S1 Live-cell imaging of YFP::CESA6, RFP::TUA and merge of YFP::CESA6 and RFP::TUA in cells treated with *B. sp*. -A secretion. The movie comprises 61 frames spaced 5 s apart at the plasma membrane focal plane after 4 hr exposure to crude secretion. Scale bars  = 10 µm.
(MOV)

Movie S2 Live-cell imaging of YFP::CESA6, RFP::TUA and merge of YFP::CESA6 and RFP::TUA in cells treated with *B. sp*. -B secretion. The movie comprises 61 frames spaced 5 s apart at the plasma membrane focal plane after 4 hr exposure to crude secretion. Scale bars  = 10 µm.
(MOV)
