# Supplementary figures and images for: Acetobixan, an Inhibitor of Cellulose Synthesis Identified by Microbial Bioprospecting
Source: PLoS One. 2014 Apr 18;9(4):e95245. doi: 10.1371/journal.pone.0095245 (PMC3991599; doi:10.1371/journal.pone.0095245)

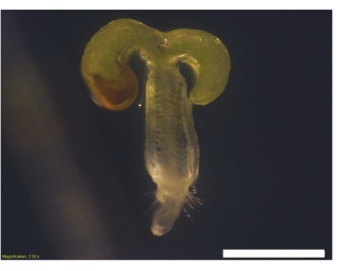

Supplement: Figure S1 — Wild type Arabidopsis seedling treated with 20 µM acetobixan for 5 days displays radial cellular swelling (scale bar = 1 mm). (JPG) [file pone.0095245.s001.jpg]
