# Supplementary material for: Acetobixan, an Inhibitor of Cellulose Synthesis Identified by Microbial Bioprospecting
Source: PLoS One. 2014 Apr 18;9(4):e95245. doi: 10.1371/journal.pone.0095245 (PMC3991599; doi:10.1371/journal.pone.0095245)
Supplement: File S1 — Outlines experimental procedures used to generate Movie S1 and S2. (DOCX) [file pone.0095245.s002.docx]

**Acetobixan, an inhibitor of cellulose synthase membrane association identified by microbial bioprospecting**

Xia Ye^†^, Lei Lei^∞^, Chad Brabham^†^, Jozsef Stork^†^, James Strickland ^Ø,1^, Adam Ladak^‡^, Ying Gu^∞^, Ian Wallace*, Seth DeBolt^†,2^

**EXPERIMENTAL PROCEDURES**

*Confocal microscopy imaging of cellulose synthase complexes*

The construction of transgenic Arabidopsis plants expressing GFP-CesA3 or YFP-CesA6 under their native promoters, dual-labeled lines expressing GFP-CesA3 and 35S:: mCherry-TUA5, and GFP-PIP2. Seeds were surface sterilized and stratified as described above and were plated on MS-agar medium lacking sucrose. Seedlings were grown vertically in the dark at 22⁰ C for 3 days. For microscopic observations, seedlings were mounted in water between 24 x 60 mm glass slides and 22 x 22 mm cover slips separated by vacuum grease spots. For drug treatments, the mounting solution was supplemented with 50 µM test compound and incubated for 1 hr in darkness at 25⁰ C prior to imaging. Seedlings treated with 0.25% (v/v) DMSO served as negative controls. The seedlings were observed using a Leica SD6000 inverted confocal microscope system featuring a 100X/ 1.4 NA oil immersion objective, a Yokogawa CSU-X1 spinning disk head, 488 and 561 nm lasers, and Metamorph control software (Molecular Devices). Z-series images were collected at a step size of 200 nm and analyzed using ImageJ software (<http://rsbweb.nih.gov/ij/>).
